# Clinical application and reporting of neurofilament quantification in neuropsychiatric disorders: an international overview

**DOI:** 10.1002/alz70856_104274

**Published:** 2025-12-24

**Authors:** Constance Delaby, Aurélie Ladang, Jose Yriarte, Chiara Zecca, Giancarlo Logroscino, Peter Koertvelyessy, Hayrettin Tumani, Piero Parchi, Isabelle Quadrio, Melanie Hart, Dorte Aalund Olsen, Daniel Alcolea, Kaj Blennow, Juan Fortea, Alberto Lleó, Alicia Algeciras‐Schimnich, Xavier Ayrignac, Aurélie Bedel, Gustavo A A Santos, Wyllians Vendramini Borelli, Elodie Bouaziz Amar, Inês Baldeiras, Edith Bigot‐Corbel, Maria Bjerke, Mercedes Carretero Perez, Tiziana Casoli, Tinatin Chabrashvili, Miles D Chapman, Jessica Cusato, Erdinc Dursun, Anthony Fourier, Daniela Galimberti, Duygu Gezen‐Ak, Brian A. Gordon, Julien Gouju, Silvia de las Heras Florez, Juan José Hernández Sánchez, Marina Herwerth, Daniela Imperiale, Flora Kaczorowski, Kensaku Kasuga, Ashvini Keshavan, Michael Khalil, Jens Kuhle, Christoph Leithner, Piotr Lewczuk, Franck Letournel, Magda Tsolaki, Guido Maria Giuffrè, Marie‐Céline Blanc, Barbara Mroszko, Jose Rodríguez‐Álvarez, Giulia Musso, Agnieszka Kulczynska‐Przybik, Léonor Nogueira, Claire Paquet, Simone Baiardi, Lorenzo Gaetani, Lucilla Parnetti, Raquel Perez Garay, Koen Poesen, Muriel Quillard‐Muraine, Enrique Rodriguez Borja, Susanna Schraen‐Maschke, Daniela Terracciano, Franziska Bachhuber, Steffen Halbgebauer, Socrates Tzartos, John Tzartos, Nadine Unterwalder, Lisa Vermunt, Cheryl L Wellington, Henrik Zetterberg, Charlotte E. Teunissen, Sylvain Lehmann

**Affiliations:** ^1^ Sant Pau Memory Unit, Hospital de la Santa Creu i Sant Pau ‐ Biomedical Research Institute Sant Pau ‐ Universitat Autònoma de Barcelona, Barcelona, Spain; ^2^ Université de Montpellier, Montpellier, France; ^3^ IRMB, INM, Université de Montpellier, INSERM, CHU de Montpellier, Laboratoire de Biochimie‐Protéomique clinique, Montpellier, France; ^4^ CHUI Liège, Liège, Liège, Belgium; ^5^ Hospital do Meixoeiro, Meixoeiro, Spain; ^6^ University of Bari “A. Moro” at Pia Fondazione “Cardinale G. Panico, Tricase, lecce, Italy; ^7^ University of Bari, Bari, Italy; ^8^ Charité – Universitätsmedizin Berlin, corporate member of Freie Universität Berlin and Humboldt‐Universität zu Berlin, Department of Neurology, Charitéplatz 1, Berlin, Berlin, Germany; ^9^ University of Ulm, Ulm, Germany; ^10^ University of Bologna, Bologna, Italy; ^11^ Centre de Recherche en Neurosciences de Lyon Inserm U1028 ‐ CNRS UMR5292 ‐ Groupement Hospitalier Est ‐ Bron, Lyon, Auvergne‐Rhône‐Alpes, France; ^12^ Department of Neuroinflammation, UCL Queen Square Institute of Neurology, London, London, United Kingdom; ^13^ University hospital of southern Denmark, Odense, southern Denmark, Denmark; ^14^ Hospital de la Santa Creu i Sant Pau, Barcelona, Barcelona, Spain; ^15^ Sant Pau Memory Unit, Hospital de la Santa Creu i Sant Pau, Institut de Recerca Sant Pau ‐ Universitat Autònoma de Barcelona, Barcelona, Spain; ^16^ Institute of Neuroscience and Physiology, Sahlgrenska Academy, University of Gothenburg, Gothenburg, Sweden; ^17^ Mayo Clinic, Rochester, MN, USA; ^18^ Montpellier university, Montpellier, Occitanie, France; ^19^ CHU Bordeaux, Bordeaux, France; ^20^ UNICAMP, Campinas, Brazil; ^21^ Brain Institute of Rio Grande do Sul (InsCer), PUCRS, Porto Alegre, Rio Grande do Sul, Brazil; ^22^ Cognitive Neurology Center, Hôpital Lariboisière‐Fernand Widal APHP, Paris, France; ^23^ Center for Innovative Biomedicine and Biotechnology, University of Coimbra, Coimbra, Portugal; ^24^ CHU Nantes, Nantes, France; ^25^ Vrije Universiteit Brussel (VUB), Brussels, Belgium; ^26^ Santa Cruz de Tenerife, Tenerife, Spain; ^27^ IRCCS INRCA, Ancona, Italy; ^28^ SUNY Upstate Medical University, Syracuse, NY, USA; ^29^ Neuroimmunology and CSF laboratory, National Hospital for Neurology and Neurosurgery, Queen Square, London, United Kingdom; ^30^ UNITO, Turin, Italy; ^31^ Department of Neuroscience, Institute of Neurological Sciences, Istanbul University‐Cerrahpasa, Istanbul, Turkey; ^32^ HCL, Lyon, France; ^33^ Fondazione IRCCS Ca’ Granda, Ospedale Maggiore Policlinico, Milan, MI, Italy; ^34^ Institute of Neurological Sciences, Istanbul University‐Cerrahpasa, Istanbul, Turkey; ^35^ Department of Radiology, Washington University School of Medicine, Saint Louis, MO, USA; ^36^ CHU Angers, Angers, France; ^37^ Hospital Universitario Nuestra Señora de Candelaria, Santa Cruz de Tenerife, Spain; ^38^ Unitat de Neuroquímica Laboratori de Referència de Catalunya, Barcelona, Spain; ^39^ USZ, Zurich, Switzerland; ^40^ Niigata University, Niigata, Niigata, Japan; ^41^ Dementia Research Centre, UCL Queen Square Institute of Neurology, University College London, London, United Kingdom; ^42^ Medical University of Graz, Graz, Austria; ^43^ University Hospital Basel, Basel, Switzerland; ^44^ Charité Universitätsmedizin Berlin, Berlin, Germany; ^45^ Department of Neurodegeneration Diagnostics, Medical University of Białystok, Białystok, Poland; ^46^ Aristotle University of Thessaloniki, Thessaloniki, Greece; ^47^ University of Padova, Padova, Italy; ^48^ APHP Site Cochin, Paris, France; ^49^ Medical University of Bialystok, Bialystok, Poland; ^50^ Centro de Investigación Biomédica en Red sobre Enfermedades Neurodegenerativas (CIBERNED), Madrid, Spain; ^51^ CHU‐PURPAN, Toulouse, France; ^52^ INSERM UMR‐S1144, Paris, France; ^53^ IRCCS Istituto delle Scienze Neurologiche di Bologna, Bologna, Italy; ^54^ Laboratory of Clinical Neurochemistry, Section of Neurology, University of Perugia, Perugia, Italy; ^55^ Biobizkaia, Bizkaia, Spain; ^56^ Laboratory for Molecular Neurobiomarker Research, Department of Neurosciences, Leuven Brain Institute, KU Leuven, Leuven, Leuven, Belgium; ^57^ Clinical Biology Center, Rouen University Hospital, Rouen, France; ^58^ Valencia, Valencia, Spain; ^59^ Univ. Lille, Lille, France; ^60^ University Federico II, Naples, Italy; ^61^ German Center for Neurodegenerative Diseases (DZNE e.V.), Ulm, Germany; ^62^ University of Athens, Athens, Greece; ^63^ Second Department of Neurology, National & Kapodistrian University of Athens, Athens, Greece; ^64^ LaborBerlin, Berlin, France; ^65^ Neurochemistry Laboratory, Department of Laboratory Medicine, Vrije Universiteit Amsterdam, Amsterdam UMC location VUmc, Amsterdam, Netherlands; ^66^ University of British Columbia, Vancouver, BC, Canada; ^67^ Department of Psychiatry and Neurochemistry, Institute of Neuroscience and Physiology, The Sahlgrenska Academy at the University of Gothenburg, Mölndal, Västra Götalands län, Sweden; ^68^ Alzheimer Center Amsterdam, Neurology, Vrije Universiteit Amsterdam, Amsterdam UMC location VUmc, Amsterdam, Netherlands; ^69^ INSERM U1183, CHU de Montpellier, Montpellier, France; ^70^ LBPC‐PPC, Université de Montpellier, IRMB CHU de Montpellier, INM INSERM, Montpellier, Occitanie, France

## Abstract

**Background:**

The quantification of Neurofilament Light chain (NfL) in blood and cerebrospinal fluid (CSF) has proven valuable for diagnosing and prognosing various neurological disorders, including Alzheimer's disease, frontotemporal dementia, amyotrophic lateral sclerosis, parkinsonism, multiple sclerosis, and ischemic damage. However, there is considerable variability in clinical and laboratory practices between centers, primarily due to different contexts of use (COU), analytical methods, and the application of cutoffs or scales. Additionally, for the same biochemical profile, the interpretation and reporting of results may vary from one center to another, raising concerns about the commutability of the tests. To date, no consensus has been reached among laboratories to define the most appropriate use of cutoffs or conclusions/comments based on NfL profiles.

**Method:**

This international project involves 38 laboratories across 18 countries, all specialized in the biochemical diagnosis of neurological disorders. Using a questionnaire, we obtained descriptions from each center regarding their COU, pre‐analytical and analytical protocols (including the biological fluid and methods used to quantify NfL). Through a consensus approach, we developed a harmonized strategy for the use and reporting of NfL results across different centres according to their respective COU.

**Results:**

Among the centers, 63% quantified NfL in the CSF, 87% in blood and 53% in both fluids. COU were as follow: frontotemporal dementia (71%), Alzheimer disease (71%), multiple sclerosis (61%), amyotrophic lateral sclerosis (61%), psychiatric syndrome (45%), Creutzfeldt‐Jakob disease (32%), Parkinson disease (39%), peripheral neuropathy (29%), traumatic brain injury (21%), Huntington's disease (13%), amyloid transthyretin related (11%) and cardiac arrest (8%). Most of the centers define pathological cutoffs based on publications (50%) and take age into account for this purpose (42%). Reporting is mostly transmitted through numeric results (95%).

**Conclusion:**

Harmonizing cutoffs, reporting, and interpretation across various clinical contexts will facilitate the incorporation of this biomarker into routine clinical practice.

